# Knowledge and attitude on tooth wear among Najran population, KSA

**DOI:** 10.6026/973206300181029

**Published:** 2022-10-31

**Authors:** Abdulrahman Aseri, Siraj DAA Khan, Abdulrahman M Aljarullah, Fahad H Al-aqil, Salem Almasabi, Nasser S Alqathia, Fahad F Al Haider, Yassin Mana Al Gfenh

**Affiliations:** 1 Department of Preventive Dental Sciences, Faculty of Dentistry, Najran University, KSA;

**Keywords:** Tooth wear (TW), pathological, dietary, severity, Prevention

## Abstract

Tooth wear is the loss of a hard dental surface. This condition might occur due to some external or internal factors. The physiological, as well as pathological factors also caused loss of tooth surface. The aim of the following survey was to evaluate the knowledge and attitude of the Najran population about tooth wear. A questionnaire-based study was conducted among the people of Najran. Questions regarding attitude and knowledge about tooth wear were asked from the participants. Data were collected and statistically analyzed. It has been seen that participants of both genders almost have the same knowledge about tooth wear. However, females were more concerned with their dietary habits and the use of fluoride toothpaste. The participants of all age groups were well familiar with tooth wear and its prevention. It has been clear from the results that residents of Najran have good knowledge about the causes of tooth wear and its prevention but there is a need to increase their practices and change their food habits to avoid the severity of Tooth wear.

## Background:

Tooth wear is one of the most severe dental conditions which increased the risk of irreversible loss of normal dental tissues, especially hard tissues that lose their intact with teeth and hence caused certain oral limitations. [1, 2] Erosive tooth wear is the most lethal and harmful condition which is caused by different factors such as intrinsic which includes the gastric reflux and excessive vomiting and extrinsic which includes the acidic foods and drinks and acid fumes. In such conditions at an early stage, patients usually don't feel the clinical symptoms while on the other hand, in later stages, erosion increased the risk of damaging the hard dental tissues and other serious medical conditions observed such as tooth hypersensitivity, the high abundance of loss of occlusal vertical height and pulp. [3] Tooth surface loss (TSL), or tooth wear (TW) is the loss of dental hard tissue that is characterized by the interaction with normal dental tissues that leads to the destruction of overall teeth structure. [4] TSL is considered a physiological or pathological condition and physiological TSL occurs as a result of mastication. Physiological TSL is also caused by friction between teeth with close intact combinations. [5] Erosive tooth wear is one of the most versatile dental conditions that are characterized through the impact of intrinsic and well as extrinsic acids [6] on hard dental tissues. Extrinsic acids that are the most toxic and lethal form arise from the ingestion of toxic food, including juices or drinking of soft drinks as well as sports drinks; while on the other hand, intrinsic acids that make the mouth unpleasant as the result of the egestion or vomiting. [7] Some case and experimental studies showed that inadequate usage of toxic acids especially the carbonated and most toxic beverages may cause tooth wear in elderly patients. [8, 9] It has been seen that chemical components present in the food have more effects on the teeth of young people. [8, 10] Some biological and medical remedies have used the protection. These remedies are protective factors in the form of fluoride toothpaste and biological factors for demisting the saliva as it is directly contacted with teeth and helps in chewing and biting the hard items. [11, 12] Acidic compounds as well as compounds that cause acidity significantly lower the function of soft dental tissues and ultimately lead to tooth surface loss. [13, 14] It was observed that there is a high rate of the occurrence of erosive tooth wear and it had seen in case of patients for treatment of dental repair in young people. [15, 16] It was estimated that this rate of tooth wear is about 30.4% in the case of children and adolescents aged 8 to 19 years. [9] There is a strong relationship between dietary acids and tooth wear that makes the compact surface with the dental tissues and declines their softness. [17, 18] In 2015, it was estimated that 13.3 billion liters of soft drinks were consumed as regular drinks in well-developed countries like the UK with an average intake of 203.6 liters per capita. [19] In some countries, it has been observed that fruit flavorings had been increased in large quantities. [19] While, excessive use of cola has also increased in the world due to the high demand for fast food items. [20] People thought that small fruit slices when added to water may decrease the erosive potential. [21] It was studied that excessive use of sweets, lozenges or medications has also increased the risk of the large erosive potential when regularly used in the form of cooking. It was reported that some well-developed countries have a wide range of food, drinks and potential use of medications as the erosive potential that can cause serious organ damage. These include acidic items, and other fruits terms such as bananas and peaches being on the lower end of the acid spectrum. [22] Children must have a good dental attitude and enough knowledge for the protection of teeth from tooth wear. They learn oral hygiene habits from their parents. A study which was carried out in Norway demonstrated that young adults (aged 18-20) have 88-93% knowledge about tooth wear. [23] Another major clinical-based observational study in Northwest England showed that among 105 patients aged 21-78 years, only one-third of participants answered correctly. [24] The major aims of this survey were to (1) evaluation of the knowledge about tooth wear in residents of the Najran community under different age groups and (2) to find their attitude towards the causes and prevention of tooth wear.

## Material and Methods:

A questionnaire-based study was carried out the evaluation of attitude and knowledge about tooth wear in the population of Najran. Based on the previous studies, reviews of literature and the experience of professionals, a questionnaire was developed. The questionnaire was divided into different sections which contain questions about demographic details of participants, their dental health, their dietary habits, their attitude and knowledge about tooth wear. The purpose of the survey was explained to all the participants and their consent was taken. Collected data was arranged and statistically analyzed in terms of frequency. A Chi-square test was applied to check the significance of the collected data.

### Inclusion and exclusion criteria:

People from the Najran community were included in this survey and no one is exempt fromparticipating in this study from the Najran population except those who did not give their consent.

## Results:

A total of 612 people from Najran including 296 females and 316 males participated in this survey. These participants were divided into three different age groups, i) Below 30 years ii) 31-60 years and iii) above 61 years (Table 1 - see PDF). 135 (22.1%) females and 106 (17.3%) male participants said that they have sensitive teeth. A total of 164 (26.8%) people (63 females and 101 males) admitted that their teeth become shorter and less noticeable. The number of participants who felt their teeth flat were 67 females (10.9%) and 76 male (12.4%). only 165 (27.0%) participants out of 612 experienced creaking and grinding of teeth. A total of 246 (40.2%) people including 116 females and 130 males did notice a change in their teeth bite while 366 (59.8%) did not notice this type of change. 69 female participants and 49 male participants said that they feel pain and tightness of the face after waking up while the remaining participants did not experience it. 39 female and 51 male participants (6.4% and 8.3 % respectively) said that their family members noticed teeth gnashing while sleeping. Half responses (pain and tightness in the face, sensitive teeth and shorter teeth) were significant (P<0.05) while other questions showed a non-significant responses (Table 2 - see PDF). Participants answered the questions about their food habits. Among 612 participants 289 (47.2%) agreed that their food contain acids and citrus fruits while 323 (52.8%) people denied it. A large number of participants (85.6%) said that they did not leave food and drinks in their mouth before swallowing and only 88 (14.4%) people agreed about these habit. 494 (80.7%) participants out of 612 did not use acidic drinks before going to bed and 118 (19.3%) use acidic drinks. Only 110 people (18.0%) have a habit of drinking sports drinks during exercise. 299 (48.9%) participants said they wash their mouths immediately after drinking cola drinks and 313 (51.1%) don't wash their mouths. Only 109 (17.8%) participants brush their teeth after drinking juice and 189 (30.9%) people agreed that they use juice lollipops when drinking soft drinks. All the answers were significant (P<0.05) except for the last two responses (Table 3 - see PDF). 88 females and 92 males said that their mouths remain always dry. About the question of any systematic disease, 68 % of participants (46 females and 22 males) said that they have an eating disorder, 61 (10.0%) participants (24 females and 37 males) had stomach reflux while the others (78.9%) did not have any systemic disease. A few number of participants (9.0%) including 25 females and 30 males worked in acidic environments. Most of the participants (211 females and 207 males) did not have a habit of biting on hard objects while 194 (31.7%)participants had this habit. 11 (1.8%), 131 (21.4%), 101 (16.5%) female participants said that they use hard, soft and medium toothbrush respectively while 53 (8.7%) didn't know the type of toothbrush. Among male participants, 13 (2.1%), 129 (21.1%) and 89 (14.5%) said that they use hard, soft and medium toothbrushes respectively and 85 (13.9%) didn't know the toothbrush type. A total of 452 (73.9%) participants used fluoride-containing toothpaste and 359 (58.7%) agreed that they care about using fluoride toothpaste. Some responses were significant (P<0.05 and P<0.01) whereas some questions (dry mouth, work in an acidic environment, biting habit) had a non-significant response (Table 4 - see PDF). About the knowledge of tooth erosion, 279 (45.6%) females and 269 (44.0%) males thought that this problem needs treatment while a few participants (17 females and 47 males) didn't think that tooth erosion is a problem. 260 (42.5%) female participants and 248 (40.5%) male participants said that they will go to the dentist in case of erosion of teeth. 267 females (43.6%) agreed that they will change their eating and behavioral habits to prevent tooth erosion. The number of male participants for the same responses was 252 (41.2%) and 263 (43.0%) respectively. A large number of people (95.8%) agreed that dental health is as important as general health. All responses were significant (P<0.05) (Table 5 - see PDF). Among 241 (39.4%) participants who had sensitive teeth, 117 (19.1%) were below 30 years, 120 (19.6%) were 31-60 years and 4 (0.7%) participants were above 61 years. The people who noticed that their teeth become shorter included 82 (13.4%), 78 (12.7%) and 4 (0.7) participants from below 30, 31-60 and above 61 years respectively.68 (11.1%) people aged below 30 years, 75 (12.3%) from 31-60 years felt their teeth flat while the people above 61 years didn't feel their teeth flat. Most of the people 264 (below 30 years) and 182 (31-60 years) didn't experience creaking and grinding of teeth. While most people above 61 years experienced it. Mostly the participants from all age groups didn't notice a change in the bite of teeth. Similarly, the majority of the people from each group (282, 209 and 3) didn’t feel pain or tightness of the face after waking up. A similar response was found in another question where people were asked about teeth gnashing while sleeping. All answers exhibited a significant (P<0.05) effect except the response about pain or tightness and teeth-gnashing that showed a non-significant effect (Table 6 - see PDF) 164 (26.8%) participants of age below 30 years, 124 (20.3%) from 31-60 years and only 1 person above 61 years confirmed that their food contains acids and citrus fruits. Only a small number of participants from each group (8.7%, 5.4% and 0.3% respectively) had the habit of leaving drinks in their mouths for a long time before swallowing. Most of the participants 494 (80.7%) didn't drink acidic drinks before going to bed. 272 (44.4%) from below 30 years, 227 (37.1%) from 31-60 years and 3 persons of age above 61 years said that they have a habit of drinking sports drinks during exercise. 48.9% of the participants said that they wash their mouths after drinking cola drinks while 51.1% denied it. Very less number of participants from each age group (69 from below 30 years, 40 from 31-60 years and 2 from above 61 years) had a habit of tooth brushing after drinking juice. All responses for the comparison between age groups and food habits showed a non-significant effect except the response about energy drinks (Table 7 - see PDF). Only 1 person of age above 61 years and 107 (17.5%) people below 30 years thought that their mouth is always dry. 31, 37 participants had eating disorders from the group below 30 years and 31-60 years respectively. 13 (2.1%), 46 (7.5%) and 2 (0.3%) participants (from below than 30, 31-60 and above 61 years respectively) had stomach reflux. A few numbers of participants (28 below 30, 24 from 31-60 and 3 above 6. years) worked in an acidic environment. 128 (20.9%), 63 (10.3%) and 3 (0.5%) participants from below 30, 31-60 and above 61 years respectively had a habit of biting on hard objects. From the age group below 30 years, 138 (22.5%) participants used hard toothbrushes, from the group of 31-60 years 119 used soft toothbrushes while 3 (0.5%) participants used soft toothbrushes. Most of the participants from each age group used fluoride-containing toothpaste and similarly a large number of people did care about using fluoride toothpaste. Most of the responses were significant (P<0.05) except the response about the type of toothbrush and dry mouth (Table 8 - see PDF). A total number of 305 (49.8%) from the group below 30 years, 238 (38.9%) from 31-60 years and all the participants of age above 61 years thought that tooth erosion is a problem that needs treatment. The majority of the participants agreed that they will go to the dentist if their teeth will be eroded. Overall, 519 (84.8%) and 530 participants said that they will change their eating and behavioral habits to prevent the erosion of teeth. Responses about knowledge were not significant (Table 9 - see PDF).

## Discussion:

Tooth wear and dental erosion are most common in children and adolescents but in the researcher's community, this condition has attained less attention comparatively. A comprehensive study cleared that the knowledge gap is the main hindrance in the diagnosis of tooth wear, its prevention and treatment. [25] Therefore, it is of interest to check the knowledge about the causes and prevention of tooth wear among people of Najran using a questionnaire-based study. Gastro-esophageal reflux disease (GERD) is considered the main intrinsic risk factor for erosive tooth wear (ETW). [26] It has been reported that intake of fruit juices and fruits on regular basis is a common causative factor for tooth wear. [27] In the current study, 68 and 61 participants had eating disorders and stomach refluxes respectively. Almost 50% of the participants use food that contains a lot of acids. This could be a reason for symptoms of tooth wear in participants. In another study, incorrect answers were given by half of the respondents. It showed the knowledge gaps. However, the questions about energy and sports drinks, mineral water, cola drinks, and fruit juices were correctly answered by almost all respondents. [28] The present survey showed that participants were much aware form the food habits that can cause tooth wear. More than half of the participants had good dietary habits. More than 80 % of the participants of the current survey considered tooth wear a problem that needs treatment. And they are willing to change their eating and behavioural habit for the protection of teeth. In a similar study, 88-94% of participants knew the term tooth wear, but their effects and causes were not familiar to them. [12]

### Strengths and limitations of the study:

The inclusion of all age groups was the strength of this study. And these participants were chosen randomly. The sample size could be a limitation of this survey. It may be supposed that participants were more prevention minded.

## Conclusions:

It has been clear from the results that residents of Najran have good knowledge about the causes of tooth wear and its prevention. They considered it a dental problem. They also showed a positive attitude. Most of the participants have healthy food habits but there is a need to increase their knowledge and change their food habits (for example usage of the toothbrush after intake of acidic food) to avoid the severity of Tooth wear.
